# Transcriptomic characteristics according to tumor size and SUV_max_ in papillary thyroid cancer patients

**DOI:** 10.1038/s41598-024-61839-0

**Published:** 2024-05-14

**Authors:** Sang-Hyeon Ju, Seong Eun Lee, Shinae Yi, Na Rae Choi, Kun Ho Kim, Seong Min Kim, June-Young Koh, Seon-Kyu Kim, Seon-Young Kim, Jun Young Heo, Junyoung O. Park, Seongyeol Park, Bon Seok Koo, Yea Eun Kang

**Affiliations:** 1 Division of Endocrinology and Metabolism, Department of Internal Medicine, Chungnam National University Hospital and College of Medicine, Daejeon, Republic of Korea; 2 Research Center for Endocrine and Metabolic Disease, Research Institute for Medical Sciences, College of Medicine, Chungnam National University, Daejeon, 35015 Republic of Korea; 3 Department of Nuclear Medicine, Chungnam National University Hospital and College of Medicine, Daejeon, Republic of Korea; 4 GENOME INSIGHT THECNOLOGY Inc, Daejeon, 35015 Republic of Korea; 5 Personalized Genomic Medicine Research Center, Korea Research Institute of Bioscience and Biotechnology, Daejeon, Republic of Korea; 6 Korea Bioinformation Center, Korea Research Institute of Bioscience and Biotechnology, Daejeon, Republic of Korea; 7 Department of Biochemistry, College of Medicine, Chungnam National University, Daejeon, Republic of Korea; 8 Department of Chemical and Biomolecular Engineering, University of California, Los Angeles, Los Angeles, CA USA; 9 Department of Otolaryngology-Head and Neck Surgery, Chungnam National University Hospital and College of Medicine, Daejeon, 35015 Republic of Korea

**Keywords:** Papillary thyroid carcinoma, SUV_max_, PET/CT, Transcriptomics, Cancer metabolism, Thyroid cancer, Cancer, Genetics, Molecular biology, Diseases, Endocrinology, Medical research

## Abstract

The SUV_max_ is a measure of FDG uptake and is related with tumor aggressiveness in thyroid cancer, however, its association with molecular pathways is unclear. Here, we investigated the relationship between SUV_max_ and gene expression profiles in 80 papillary thyroid cancer (PTC) patients. We conducted an analysis of DEGs and enriched pathways in relation to SUV_max_ and tumor size. SUV_max_ showed a positive correlation with tumor size and correlated with glucose metabolic process. The genes that indicate thyroid differentiation, such as *SLC5A5* and *TPO,* were negatively correlated with SUV_max_. Unsupervised analysis revealed that SUV_max_ positively correlated with DNA replication(r = 0.29, *p* = 0.009), pyrimidine metabolism(r = 0.50, *p* < 0.0001) and purine metabolism (r = 0.42, *p* = 0.0001). Based on subgroups analysis, we identified that *PSG5*, *TFF3*, *SOX2*, *SL5A5*, *SLC5A7*, *HOXD10*, *FER1L6*, and *IFNA1* genes were found to be significantly associated with tumor aggressiveness. Both high SUV_max_ PTMC and macro-PTC are enriched in pathways of DNA replication and cell cycle, however, gene sets for purine metabolic pathways are enriched only in high SUV_max_ macro-PTC but not in high SUV_max_ PTMC. Our findings demonstrate the molecular characteristics of high SUV_max_ tumor and metabolism involved in tumor growth in differentiated thyroid cancer.

## Introduction

Thyroid cancer accounts for the largest portion of endocrine cancers, and its incidence has been increasing in the past decades, with 586,000 cases in 2020 worldwide^1^. In papillary thyroid cancer (PTC), accounting for 80–85% of all thyroid cancers, current disease staging and therapeutic decision-making are primarily based on tumor size and node metastases^2^. However, not all cases follow the predicted disease course upon initial staging. For example, during 3 to 10 years of active surveillance for papillary thyroid microcarcinoma (PTMC), which is defined as a tumor of 1.0 cm or less in size, 3.5–14.1% of tumors were found to increase in size by > 3 mm, and up to 1.5% of cases progressed to lymph node metastasis^3^. Although clinical studies suggest that young age, lymph node metastasis, local invasion to the nerve or trachea, and high-grade malignancy on cytology are risk factors for progression during active surveillance^3,4^, there is a lack of molecular genetic markers to explain why some tumors progress rapidly^5^.

Although researchers have sought to establish a relationship between *BRAF*^V600E^ and thyroid cancer diagnosis or prognosis, the relationship remains inconclusive^6–8^. More recently, the *TERT* promoter mutation has been found to coexist with the *BRAF*^V600E^ mutation, which is recognized as an indicator of clinically aggressive tumors^9–11^; however, the prevalence of patients with both mutations is low, and there seems to be no correlation with prognosis, especially in PTMC^12,13^. Large-scale genomic characterization of PTC uncovered the critical role of genetic alterations that activate the mitogen-activated protein kinase (MAPK), including *BRAF* and *RAS*; as well as, gene fusions of protein kinase genes such as *RET*, *NTRK1*, and *NTRK3* as well as mutations in phosphoinositide 3-kinase AKT (PI3K-AKT) pathways including *PTEN*, *PI3KCA*, and *AKT1* were identified as the primary molecular aberrations in PTC^14,15^. However, somatic mutation profiling is still insufficient for risk stratification of patients with PTC.

^18^F-fluorodeoxyglucose (FDG) PET/CT is increasingly performed for the staging or localization of metastatic disease in patients with various kinds of malignancies^16,17^. In thyroid cancer, iodine-131-WBS has been useful for determining the differentiation of a tumor on the basis of its avidity to iodine, identifying remnant thyroid tissue, and assessing patients for distant metastatic disease^18^; most well-differentiated thyroid carcinomas are relatively slow growing and can be FDG-negative^19^. Recently, however, a “flip-flop phenomenon” has been observed, in that radioiodine uptake in differentiated thyroid carcinoma (DTC) cells decreases when they dedifferentiate while their glucose metabolism generally increases, and ^18^F-FDG PET/CT has emerged as a powerful tool for predicting recurrence in DTC patients^20^. Moreover, preoperative SUV_max_ was found to be related to postoperative recurrence-free survival (RFS)^21^. It is commonly accepted that a high ^18^F-FDG uptake reflects the dedifferentiation of thyroid tumors; however, there is little data on the role of ^18^F-FDG PET/CT in thyroid cancer, and the underlying molecular glucose metabolism mechanisms are not entirely understood.

This study is the first to report findings on ^18^F-FDG PET/CT in thyroid cancer in conjunction with a gene expression analysis in an attempt to examine the molecular characteristics related to metabolism in thyroid cancer. We investigated the relationship between SUV_max_ and clinical features of PTC; we also analyzed differentially expressed genes (DEGs) and activated pathways related to SUV_max_. The significance of genes and pathways were validated using The Cancer Genome Atlas (TCGA) database.

## Results

### Enriched DNA replication, pyrimidine and one-carbon metabolism, and cell cycle signaling with reduced thyroid differentiation scores (TDS) in PTCs with high SUV_max_

**Figure 1:**
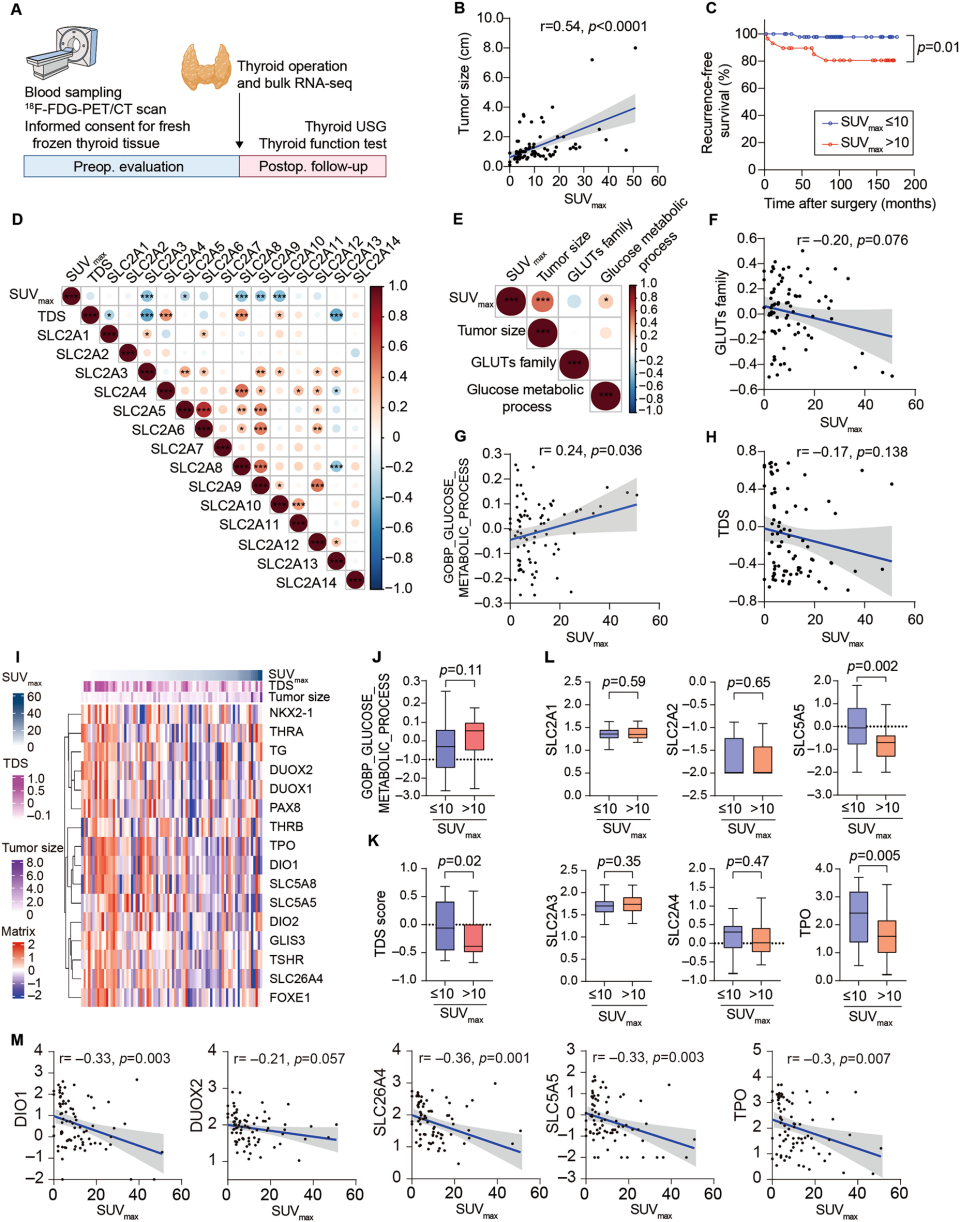
SUV_max_ were not significantly correlated with GLUTs or glycolysis, however, PTCs with high SUV_max_ are enriched low TDS score than those with low SUV_max_. (**A**) The design of our studies to verify the metabolic features by SUV_max_ in PTC (N = 80). (**B**) The correlation plot between tumor size and SUV_max_ using Pearson correlation method (r = 0.54, *p* < 0.0001). (**C**) The recurrence-free survival probability of low SUV_max_ (n = 50, blue) and high SUV_max_ group (n = 30, red). The *p* = 0.013 is from log-rank test. (**D**) Correlation plots of SUV_max_ with GLUTs family and TDS. (**E**–**H**) Correlation plots of SUV_max_ with tumor size, TDS, GSVA score of GLUTs family, and GSVA score Glycolysis. (**I**) Heatmap of TDS genes using log_10_TPM in our cohort. Tumors were sorted according to SUV_max_. (**J**,**K**) Comparison of GSVA for glycolysis or tumor differentiation score (TDS) between PTC^SUV-low^ and PTC^SUV-high^ tumors. (**L**) Expression of genes encoding SLC2A1, SLC2A2, SLC2A3, SLC2A4, SLC5A5, and TPO between PTC^SUV-low^ and PTC^SUV-high^ tumors. (**M**) Expression of TDS genes, *DIO1*, *DUOX2*, *SLC26A4*, *SLC5A5* and *TPO*, between PTC^SUV-low^ and PTC^SUV-high^ tumors, and scatter plots of correlation between SUV_max_ and either *SLC5A5* or *TPO*. In the scatter plots, blue line is drawn using simple linear regression and the gray colored area indicate 95% confidence band.

All 80 patients underwent preoperative ^18^F-FDG-PET/CT (Fig. 1A). As previously reported^22,23^, SUV_max_ correlated with tumor size (r = 0.54, *p* < 0.0001) (Fig. 1B). Since the predictive preoperative SUV_max_ value for tumor recurrence derived from the ROC curves was 10.15, we divided the subjects into PTC^SUV-high^ (SUV_max_ > 10) and PTC^SUV-low^ SUV_max_ ≤ 10) group (Supplementary Fig. 1) and PTC^SUV-high^ revealed the worse prognosis in recurrence free survival analysis (Fig. 1C).

Since glucose metabolism in thyroid cancer cells is reprogrammed to enhance glucose uptake, glycolysis, and lactate synthesis^24^ and glucose uptake via glucose transporters (GLUTs) is the first step in producing energy and nucleic acids for cancer survival, we investigated the expressions of GLUTs in relation to SUV_max_. Previously, thyroid cancer cells show overexpression of hypoxia-responsive GLUT1 and GLUT3 proteins compared to normal cells^25^, however, it is not fully understood the relation of GLUTs and PET-CT SUV_max_ in PTC. We found that GLUT3, GLUT5, and GLUT8-10 were negatively correlated with SUV_max_ and othter GLUTs were not significantly associated with SUV_max_ (Fig. 1D). Since many studies have focused on the impact of TDS, and tumor differentiation rate has been shown to correlate with GLUT expression^26,27^, we also analyzed TDS, GLUTs, and glucolysis according to SUV_max_ and tumor upon the scores calculated using GSVA (Fig. 1D,E). Gene score of GLUTs family was not significantly associated with SUV_max_ (r = − 0.20, *p* = 0.078) (Fig. 1F), however, gene score of glucose metabolic process was significantly associated with SUV_max_ (r = 0.24, *p* = 0.036) (Fig. 1G). TDS was not significantly correlated with SUV_max_ (r = − 0.17, *p* = 0.138) (Fig. 1H)_,_ but The TDS expression heatmap derived from SUV_max_ showed a tendency for some of TDS gene expression were decreased in the PTC^SUV-high^ group (Fig. 1I). Although the gene score of glucose metabolic process was not significantly changed between PTC^SUV-high^ group and PTC^SUV-low^ group (Fig. 1J), PTC^SUV-high^ group revealed significantly decreased TDS score compared to PTC^SUV-low^ group (Fig. 1K). Although the levels of GLUTs genes, such as SLC2A1, SLC2A2, SLC2A3, and SLC2A4 were not changed, several TDS genes, *SLC5A5*, *TPO*, *DIO2*, and *TG*, were significantly lower in the PTC^SUV-high^ than in the PTC^SUV-low^ group (Fig. 1L and Supplementary Table 1). Moreover, SUV_max_ was negatively correlated with several TDS genes, *DIO1* (r = − 0.33, *p* = 0.003), *SLC5A5* (r = − 0.33, *p* = 0.003), and *TPO* (r = − 0.30, *p* = 0.007) expression (Fig. 1M).

**Figure 2:**
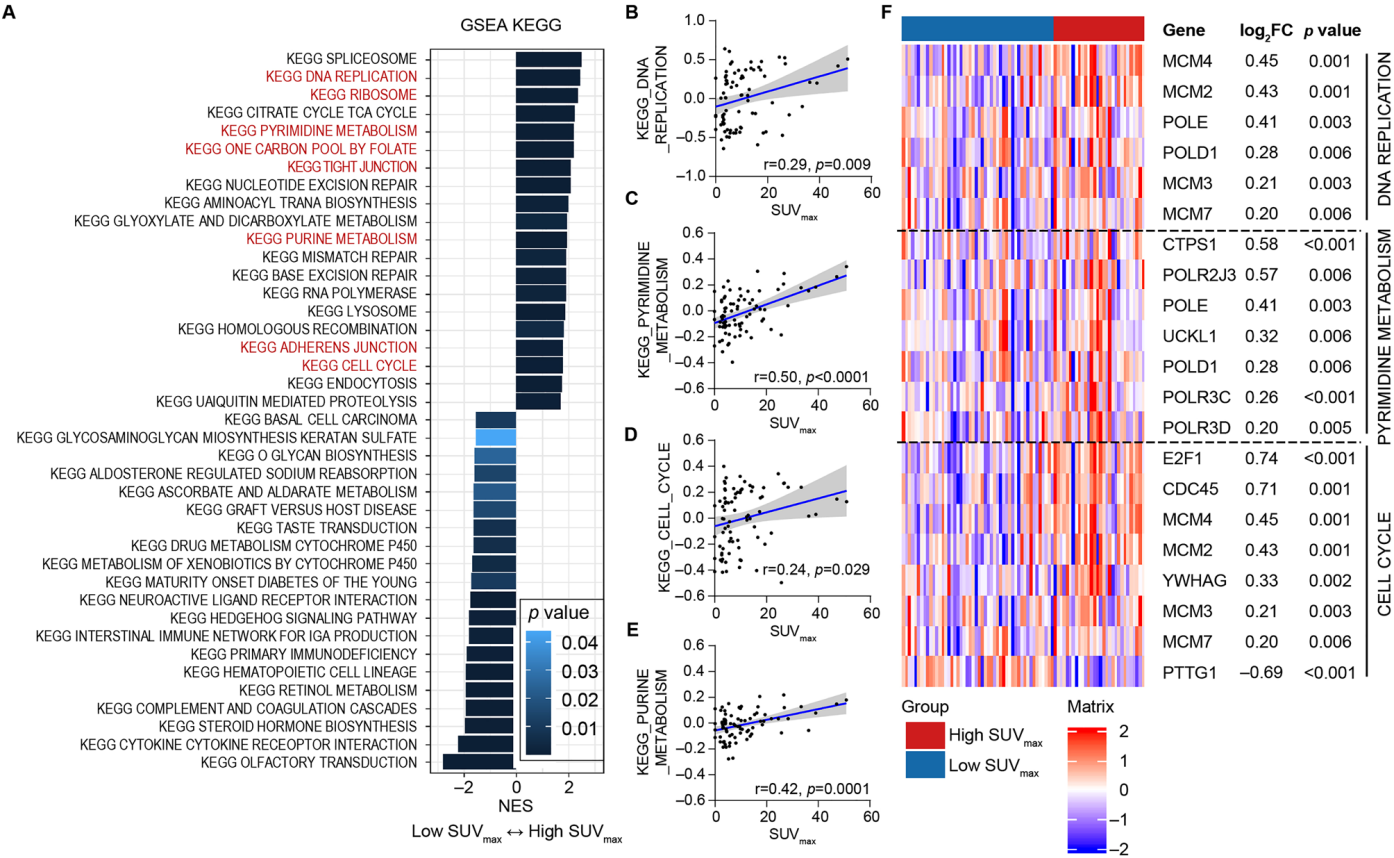
PTCs with high SUV_max_ are enriched with several pathways, such as DNA replication, cell cycle, pyrimidine metabolism, and one carbon pool by folate. (**A**) Comparison of GSVA based on unsupervised analysis using KEGG analysis between PTC^SUV-low^ and PTC^SUV-high^ tumors. (**B**–**E**) Scatter plots of correlation between SUV_max_ and GSVA score of DNA replication, pyrimidine metabolism, cell cycle, or purine metabolism. In the scatter plots, blue line is drawn using simple linear regression and the gray colored area indicate 95% confidence band. (**F**) Heatmap of genes related upregulated pathway using log_10_TPM in our cohort. Log2 fold change and p value were calculated by the comparison of gene expressions between PTC^SUV-low^ and PTC^SUV-high^ tumors.

Next, we performed unsupervised analysis based on GSEA to identify enriched pathways in each subtype. The PTC^SUV-high^ group exhibited enriched DNA replication, ribosome assembly, pyrimidine metabolism, one-carbon pool by folate, purine metabolism, tight junction, adherens junction, and cell cycle processes in the KEGG database (Fig. 2A and Supplementary Table 2). Ribosome biogenesis, DNA replication initiation, DNA-dependent DNA replication, and DNA replication from Gene Ontology Biological Process (GOBP) gene sets were also enriched in the PTC^SUV-high^ group (Supplementary Fig. 2 and Supplementary Table 3). We calculated the scores using GSVA related to various signaling pathways for each tumor sample based on the meaningful GSEA results. We confirmed a positive correlation between SUV_max_ and DNA replication (r = 0.29, *p* = 0.009), pyrimidine metabolism signaling (r = 0.50, *p* < 0.0001), cell cycle (r = 0.24, *p* = 0.029), and purine metabolism (r = 0.42, *p* = 0.0001) (Fig. 2B–E). In addition, the expression of genes related to DNA replication, one-carbon metabolism, and cell cycle was higher in the PTC^SUV-high^ group (Fig. 2F). Collectively, our data revealed that SUV_max_ of thyroid tumor was not correlated with glycolysis, but was significantly related with several molecular pathways, including DNA replication, cell cycle, pyrimidine metabolism and purine metabolism.

### Importance of SUV_max_ in predicting tumor aggressiveness after adjustment of tumor size

Since our data revealed the positive correlation of tumor size and SUV_max_, we compared to various clinic-pathologic features in relation to tumor size or SUV_max_ to investigate the pivotal gene signature in relation to SUV_max_, independently on tumor size (Table 1). In comparison of PTC^SUV-high^ and PTC^SUV-low^, PTC^SUV-high^ has more extracapsular invasion (ECI, *p* = 0.003), gross extrathyroidal extension (gross ETE, *p* = 0.013), and recurrence (*p* = 0.026) than PTC^SUV-low^. Although the PTC^SUV-high^ group had more invasive features, the distribution of subtypes and pathological stage were comparable to those in the PTC^SUV-low^ group (subtype, *p* = 0.331; pathological stage, *p* = 0.776). Next, we compared the various clinic-pathologic findings after tumor-size adjustments using ANCOVA or Mantel–Haenszel Chi-square test (Table 1). After adjustment for tumor size, PTC^SUV-high^ has also more extracapsular invasion (ECI, *p* = 0.013), gross extrathyroidal extension (gross ETE, *p* = 0.028), and recurrence (*p* = 0.047) than PTC^SUV-low^ group. These data suggested the need for establishment of the molecular features of SUV_max_ high tumors and the importance of SUV_max_ values for predicting cancer progression independent of tumor size. In addition, a comparison of the factors between PTMC and macro-PTC after adjustment to SUV_max_ revealed that lateral lymph node metastasis (L-LNM, *p* = 0.019) rate was significantly higher in macro-PTC than PTMC (Supplementary Table 4). Collectively, our clinical data suggested that large tumor was associated with lymph node metastasis after adjustment to SUV_max_, and high SUV_max_ tumor was associated with invasion or extrathyroidal extension after adjustment to tumor size.

**Table 1:** Comparisons of clinicopathologic characteristics by SUV_max_ group, unadjusted and tumor size-adjusted.

	Unadjusted			Tumor size-adjusted		
	SUV_max_ ≤ 10	SUV_max_ > 10	*p* value	SUV_max_ ≤ 10	SUV_max_ > 10	*p* value
	N = 50	N = 30		N = 50	N = 30	
Tumor size (cm)^#^	1.0 ± 0.1	1.9 ± 0.3	0.020*	–^§^	–	–
SUV_max_	4.8 ± 0.4	21.2 ± 2.0	< 0.001*	5.6 ± 0.9	19.8 ± 1.2	< 0.001*
Age	48.1 ± 1.5	45.4 ± 2.5	0.313	47.8 ± 1.7	45.9 ± 2.2	0.440
Gender (male)	4 (8%)	2 (6.7%)	0.826	4 (8%)	2 (6.7%)	0.839
BMI (kg/m^2^)	24.8 ± 0.6	25.5 ± 0.7	0.459	24.9 ± 0.6	25.3 ± 0.8	0.897
DM	4 (8%)	2 (6.7%)	0.826	4 (8%)	2 (6.7%)	0.839
Hashimoto's thyroiditis	9 (18%)	5 (16.7%)	0.879	9 (18%)	5 (16.7%)	0.669
ECI	32 (64%)	28 (93.3%)	0.003*	32 (64%)	28 (93.3%)	0.013*
Gross ETE invading only strap muscles	30 (60%)	26 (86.7%)	0.013*	30 (60%)	26 (86.7%)	0.028*
C-LNM	20 (40%)	18 (60%)	0.107	20 (40%)	18 (60%)	0.190
L-LNM	5 (10%)	8 (26.7%)	0.065	5 (10%)	8 (26.7%)	0.243
LVI	44 (88%)	30 (100%)	0.079	44 (88%)	30 (100%)	0.097
BRAF mutation	27 (54%)	20 (66.7%)	0.349	27 (54%)	20 (66.7%)	0.323
RAS mutation	3 (6%)	1 (3.3%)	0.596	3 (6%)	1 (3.3%)	0.948
Recurrence	1 (2%)	5 (16.7%)	0.026*	1 (2%)	5 (16.7%)	0.047*
Subtype, Classical	46 (92%)	25 (83.3%)	0.331	46 (92%)	25 (83.3%)	0.375
Follicular	1 (2%)	3 (10%)		1 (2%)	3 (10%)	
Tall cell	2 (4%)	2 (6.7%)		2 (4%)	2 (6.7%)	
Oncocytic	1 (2%)	0		1 (2%)	0	
Stage						
I	40 (80.0%)	25 (83.3%)	0.776	–	–	–
II	10 (20.0%)	5 (16.7%)		–	–	–

*BMI* body-mass index, *DM* diabetes mellitus, *ECI* extracapsular invasion, *ETE* extrathyroidal extension, *C-LNM* central lymph node metastasis, *L-LNM* lateral lymph node metastasis, *LVI* lymphovascular invasion, *RFS* recurrence-free survival, *F/U* follow-up. Stage was determined based on eighth edition AJCC cancer staging manual. ^#^Not adjusted tumor size is shown. *p value < 0.05. In continuous variables, unpaired *t*-test is used for unadjusted comparisons and analysis of covariance (ANCOVA) is used for tumor-size adjusted comparisons, and data are presented as mean ± SEM. ^§^Covariates of ANCOVA model are evaluated by tumor size = 1.3362. In categorical variables, Chi-square test and Mantel–Haenszel Chi-square test were used for unadjusted and tumor size-adjusted RFS comparison, respectively, and the data are presented as number (proportion in %).

To identify the major contributory molecular characteristics according to tumor size and SUV_max_ in PTC, we divided tumors into PTMC and macro-PTC by 1 cm of the tumor size and further subgrouped using 10 of SUV_max_: PTMC^SUV-low^, PTMC^SUV-high^, macro-PTC^SUV-low^, and macro-PTC^SUV-high^ (Supplementary Fig. 1B and Table 2). Consistently with the findings of Table 1, the PTMC^SUV-high^ group exhibited more ECI and gross ETE than the PTMC^SUV-low^ group (Table 2). Moreover, the PTMC^SUV-high^ group exhibited even more gross ETE than the macro-PTC^SUV-low^ group and the ECI and gross ETE rates did not significantly differ between the two SUV-high groups (PTMC^SUV-high^ and macro-PTC^SUV-high^), suggesting the importance of SUV_max_ on gross ETE, independently on tumor size. In contrast, significantly less lateral LNM (L-LNM) was observed in the PTMC^SUV-high^ group than that in the macro-PTC^SUV-high^ group (Table 2). Taken together, SUV_max_ and tumor size were independently correlated with different clinical factors, such as ETE or lymph node metastasis.

**Table 2:** Comparison of clinicopathologic characteristics of PTC by SUV_max_ and tumor size.

	① PTMC^SUV-low^	② PTMC^SUV-high^	③ Macro-PTC^SUV-low^	④ Macro-PTC^SUV-high^	① vs. ②	② vs. ③	② vs. ④
	N = 37	N = 11	N = 13	N = 19	*p* value	*p* value	*p* value
Tumor size (cm)	0.7 ± 0.2	0.8 ± 0.1	2.0 ± 0.9	2.5 ± 2.0	0.175	**0.001**	**0.002**
SUV_max_	4.2 ± 2.6	13.8 ± 3.1	6.5 ± 2.3	25.5 ± 11.4	**< 0.001**	**< 0.001**	**< 0.001**
Age	48.8 ± 9.9	44.8 ± 11.0	46.2 ± 11.8	45.7 ± 15.2	0.263	0.765	0.870
Gender (male)	3 (8.1%)	0	1 (7.7%)	2 (10.5%)	0.329	0.347	0.520
BMI (kg/m^2^)	24.5 ± 3.9	23.7 ± 2.9	25.6 ± 2.4	26.4 ± 3.1	0.612	0.182	0.085
DM	3 (8.1%)	1 (9.1%)	1 (7.7%)	1 (5.3%)	0.918	0.902	0.685
Hashimoto's thyroiditis	7 (18.9%)	1 (9.1%)	2 (15.4%)	4 (21.1%)	0.661	0.642	0.626
ECI	23 (62.2%)	11 (100%)	9 (69.2%)	17 (89.5%)	**0.021**	0.098	0.520
Gross ETE invading only strap muscles	22 (59.5%)	11 (100%)	8 (61.5%)	15 (78.9%)	**0.010**	**0.041**	0.268
C-LNM	12 (32.4%)	5 (45.5%)	8 (61.5%)	13 (68.4%)	0.486	0.682	0.266
L-LNM	2 (5.4%)	0	3 (23.1%)	8 (42.1%)	0.431	0.223	**0.014**
LVI	31 (83.8%)	11 (100%)	13 (100%)	19 (100%)	0.313	0.999	0.999
BRAF mutation	19 (51.4%)	7 (63.6%)	8 (61.5%)	13 (68.4%)	0.514	0.916	0.789
RAS mutation	3 (8.1%)	1 (9.1%)	0	0	0.918	0.458	0.367
Recurrence	1 (2.7%)	1 (9.1%)	0	4 (21.1%)	0.410	0.458	0.626

*PTMC* papillary thyroid microcarcinoma, *Macro-PTC* macro papillary thyroid carcinoma, *BMI* body-mass index, *DM* diabetes mellitus, *ECI* extracapsular invasion, *ETE* extrathyroidal extension, *C-LNM* central lymph node metastasis, *L-LNM* lateral lymph node metastasis, *LVI* lymphovascular invasion, *RFS* recurrence-free survival, *F/U* follow-up. *p value < 0.05 in comparison of PTMC^SUV-high^ and macro-PTC^SUV-low^. ^†^*p* value < 0.05 in comparison of PTMC^SUV-low^ and PTMC^SUV-high^. ^‡^*p* value < 0.05 in comparison of PTMC^SUV-high^ and macro-PTC^SUV-high^. Data are presented as mean ± SD for continuous variables and number (proportion in %) for categorical variables. Significant values are in bold.

### DEG and GSEA analyses reveal enriched metabolic pathways in PTMC^SUV-high^ and macro-PTC^SUV-high^ and new genes associated with PTC recurrence

**Figure 3:**
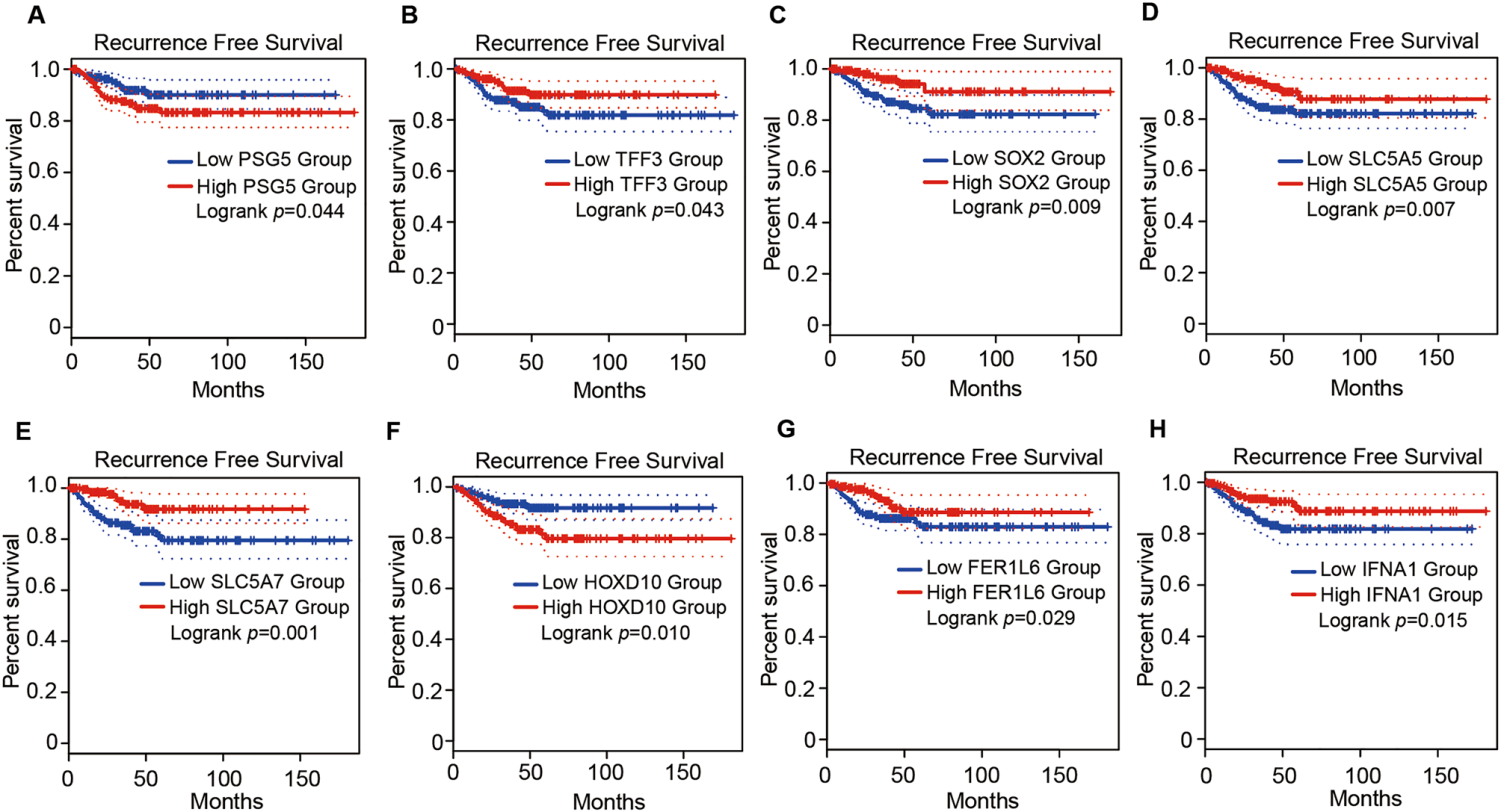
Survival plots of genes contributing RFS difference by SUV_max_ and tumor size. (**A**–**E**) Kaplan–Meier plots showing RFS difference from TCGA dataset by expression level of *PSG5* (**A**), *TFF3* (**B**), *SOX2* (**C**), *SLC5A5* (**D**), and *SLC5A7* (**E**) that contribute RFS difference by SUV_max_. The high expression of *PSG5* (**A**) and low expression of *TFF3* (**B**), *SOX2* (**C**), *SLC5A5* (**D**), and *SLC5A7* (**E**) have shorter RFS. (**F**–**H**) Kaplan–Meier plots showing RFS difference from TCGA dataset by expression level of *HOXD10* (**F**), *FER1L6* (**G**), *IFNA1* (**H**) which were found to contribute to RFS difference by tumor size. The high expression of *HOXD10* (**F**) and low expression of *FER1L6* (**G**) and *IFNA1* (**H**) have shorter RFS.

We conducted an analysis of DEGs and enriched pathways between subgroups: (1) PTMC^SUV-low^ vs. PTMC^SUV-high^, (2) macro-PTC^SUV-low^ vs. macro-PTC^SUV-high^, and (3) all SUV_max_-low vs. SUV_max_-high (Supplementary Tables 5–7, 8–10, and 1–3, respectively). The relationship between up- and down- regulated DEGs in each comparison is shown in Supplementary Fig. 3A,B. To identify DEGs related to high SUV_max_ independent of tumor size, we intersected all three comparisons and found 28 common DEGs (6 upregulated and 22 downregulated) (Supplementary Fig. 3A,B, Supplementary Table 11). Among these, one upregulated DEG (*PSG5*) and four downregulated DEGs (*TFF3*, *SOX2*, *SLC5A5*, *SLC5A7*) showed a significant difference in RFS in the GEPIA 2 database (Table 3, Fig. 3A–E). In addition, DEGs from comparison (3) that did not belong to those from comparison (1) or (2) were defined as DEGs related to SUV_max_ but not tumor size. We identified 16 upregulated DEGs and 60 downregulated DEGs (Supplementary Fig. 3A,B). Among these DEGs, one upregulated (*HOXD10*) and two downregulated (*IFNA1* and *FER1L6*) DEGs exhibited a significant difference in RFS in the GEPIA 2 database (Table 3, Fig. 3F–H). Based on these results, we identified the up- and down- regulated DEGs related to SUV_max_ or tumor size and further selected some genes associated with RFS.

**Table 3:** Selected DEGs that showed difference in RFS.

Genes	*p* value for median DFS	SUV_max_ ≤ 10 vs. SUV_max_ > 10		PTMC^SUV-low^ vs. PTMC^SUV-high^		Macro-PTC^SUV-low^ vs. PTMC^SUV-high^	
		Log_2_FC	*p* value	Log_2_FC	*p* value	Log_2_FC	*p* value
SUV_max_-related genes							
Up-regulated genes							
PSG5	0.044*	1.64	0.005*	2.11	0.028*	1.94	0.020*
Down-regulated genes							
TFF3	0.043*	− 3.05	< 0.001*	− 2.27	0.003*	− 1.87	0.014*
SOX2	0.009*	− 2.42	< 0.001*	− 2.04	0.030*	− 2.21	0.006*
SLC5A5	0.007*	− 1.8	0.004*	− 1.92	0.030*	− 3.16	0.001*
SLC5A7	0.007*	− 3.55	< 0.001*	− 2.94	0.005*	− 3.41	0.012*
Tumor size-related genes							
Up-regulated genes							
HOXD10	0.010*	1.63	0.010*	− 0.40	0.663	− 0.56	0.490
Down-regulated genes							
IFNA1	0.015*	− 1.68	0.025*	− 2.21	0.052	− 1.79	0.097
FER1L6	0.029*	− 2.15	< 0.001*	− 1.44	0.094	− 1.47	0.042*

*DFS* disease-free survival, *PTMC* papillary thyroid microcarcinoma, *Macro-PTC* Macro (> 1 cm) papillary thyroid carcinoma, *DEG* differentially expressed genes, *FC* Fold Change; *p value < 0.05 with significant fold change (Log_2_FC > 1.5 for up-regulated genes and < − 1.5 for down-regulated genes).

Our gene ontology analyses identified several gene sets that were shared across the comparisons. The distribution of upregulated KEGG and GOBP genes is shown in the Venn diagrams in Supplementary Fig. 3C,D. In KEGG analyses, genes pivotal for tumor survival and progression were enriched in PTC^SUV-high^ compared to PTC^SUV-low^ (Table 4). Our subgroup analyses revealed enriched DNA replication, cell cycle processes, and ribosome assembly in the SUV_max_-high groups compared to the SUV_max_-low groups. Interestingly, the gene sets associated with glucose metabolism, such as glycolysis, the citrate cycle (TCA cycle), and the glycolysis offshoot pathway including purine metabolism and the pentose phosphate pathway were enriched in high SUV_max_ tumors of macro-PTCs but not in those of PTMCs (Table 4). GOBP analyses also revealed that gene sets for DNA replication, cell cycle processes, and ribosome assembly were enriched in the high SUV_max_ subgroups of both PTMC and macro-PTC. In contrast, gene sets for purine metabolism, glucose import, metabolic processes, and ribose phosphate metabolism were enriched only in high SUV_max_ tumors of macro-PTCs but not in those of PTMCs (Table 5). These results suggest that purine metabolism, the ribose phosphate pathway, and glucose import may be related to tumor growth rather than SUV_max_.

**Table 4:** Up-regulated KEGG in relation to SUV_max_ and tumor size.

KEGG ID	KEGG term	SUV_max_ ≤ 10 vs. SUV_max_ > 10		PTMC^SUV-low^ vs. PTMC^SUV-high^		Macro-PTC^SUV-low^ vs. Macro-PTC^SUV-high^	
		Fold change	*p* value	Fold change	*p* value	Fold change	*p* value
hsa03030	DNA REPLICATION	2.45	< 0.001*	2.13	< 0.001*	2.49	< 0.001*
hsa04110	CELL CYCLE	1.78	< 0.001*	1.25	0.044*	1.81	< 0.001*
hsa03010	RIBOSOME	2.36	< 0.001*	2.45	< 0.001*	2.78	< 0.001*
hsa04120	UBIQUITIN MEDIATED PROTEOLYSIS	1.69	0.001*	1.71	< 0.001*	1.41	0.020*
hsa04142	LYSOSOME	1.85	< 0.001*	1.69	< 0.001*	1.91	< 0.001*
hsa04520	ADHERENS JUNCTION	1.79	0.001*	1.79	< 0.001*	1.15	0.204
hsa04530	TIGHT JUNCTION	2.07	< 0.001*	1.71	< 0.001*	1.91	< 0.001*
hsa04910	INSULIN SIGNALING PATHWAY	1.63	0.002*	1.30	0.024*	1.40	0.021*
hsa00520	AMINO SUGAR AND NUCLEOTIDE SUGAR METABOLISM	1.58	0.023*	1.57	0.008*	1.55	0.018*
hsa00010	GLYCOLYSIS GLUCONEOGENESIS	1.47	0.029*	− 1.31	0.084	2.29	< 0.001*
hsa00020	CITRATE CYCLE TCA CYCLE	2.24	< 0.001*	− 0.90	0.632	2.76	< 0.001*
hsa00240	PYRIMIDINE METABOLISM	2.19	< 0.001*	1.50	0.004*	2.24	< 0.001*
hsa00230	PURINE METABOLISM	1.94	< 0.001*	1.15	0.103	1.99	< 0.001*
hsa00670	ONE CARBON POOL BY FOLATE	2.19	< 0.001*	1.04	0.397	2.07	< 0.001*
hsa00030	PENTOSE PHOSPHATE PATHWAY	1.61	0.037*	0.99	0.472	2.13	< 0.001*

*KEGG* KEGG Kyoto Encyclopedia of Genes and Genomes, *SUV*_*max*_ maximum standardized uptake value, *PTMC* papillary thyroid microcarcinoma, *Macro-PTC* macro papillary thyroid carcinoma. *p value < 0.05.

**Table 5:** Up-regulated gene ontology (GO) biological process (GOBP) in relation to SUV_max_ and tumor size.

GO ID	GO Term	SUV_max_ ≤ 10 vs. SUV_max_ > 10		PTMC^SUV-low^ vs. PTMC^SUV-high^		Macro-PTC^SUV-low^ vs. Macro-PTC^SUV-high^	
		Fold change	*p* value	Fold change	*p* value	Fold change	*p* value
GO:0006260	DNA REPLICATION	2.38	< 0.001*	1.73	< 0.001*	2.26	< 0.001*
GO:0006270	DNA REPLICATION INITIATION	2.45	< 0.001*	1.89	0.001*	2.46	< 0.001*
GO:1902292	CELL CYCLE DNA REPLICATION INITIATION	1.90	0.001*	1.71	0.010*	1.84	0.001*
GO:1902969	MITOTIC DNA REPLICATION	2.00	0.001*	1.74	0.015*	2.10	< 0.001*
GO:0006261	DNA DEPENDENT DNA REPLICATION	2.43	< 0.001*	1.63	< 0.001*	2.38	< 0.001*
GO:0006302	DOUBLE STRAND BREAK REPAIR	1.88	< 0.001*	1.42	< 0.001*	1.75	< 0.001*
GO:0006413	TRANSLATIONAL INITIATION	2.04	< 0.001*	1.37	0.018*	2.41	< 0.001*
GO:0044770	CELL CYCLE PHASE TRANSITION	1.92	< 0.001*	1.52	< 0.001*	1.58	< 0.001*
GO:1901987	REGULATION OF CELL CYCLE PHASE TRANSITION	1.86	< 0.001*	1.53	< 0.001*	1.56	< 0.001*
GO:0044772	MITOTIC CELL CYCLE PHASE TRANSITION	1.84	< 0.001*	1.58	< 0.001*	1.59	< 0.001*
GO:0007346	REGULATION OF MITOTIC CELL CYCLE	1.84	< 0.001*	1.43	< 0.001*	1.59	< 0.001*
GO:1901990	REGULATION OF MITOTIC CELL CYCLE PHASE TRANSITION	1.77	< 0.001*	1.55	< 0.001*	1.58	< 0.001*
GO:0000723	TELOMERE MAINTENANCE	1.98	< 0.001*	1.48	0.003*	1.94	< 0.001*
GO:0032204	REGULATION OF TELOMERE MAINTENANCE	1.90	< 0.001*	1.42	0.017*	1.70	0.001*
GO:0010833	TELOMERE MAINTENANCE VIA TELOMERE LENGTHENING	1.85	< 0.001*	1.38	0.027*	1.77	< 0.001*
GO:0042254	RIBOSOME BIOGENESIS	2.73	< 0.001*	1.90	< 0.001*	2.77	< 0.001*
GO:0009127	PURINE NUCLEOSIDE MONOPHOSPHATE BIOSYNTHETIC PROCESS	1.97	0.001*	1.47	0.040*	1.81	0.003*
GO:0072521	PURINE CONTAINING COMPOUND METABOLIC PROCESS	1.50	< 0.001*	− 1.14	0.140	2.06	< 0.001*
GO:0072522	PURINE CONTAINING COMPOUND BIOSYNTHETIC PROCESS	1.67	< 0.001*	− 1.00	0.478	2.16	< 0.001*
GO:0046323	GLUCOSE IMPORT	1.40	0.035*	− 0.90	0.647	1.38	0.027*
GO:0006006	GLUCOSE METABOLIC PROCESS	1.46	0.003*	− 0.76	0.952	1.72	< 0.001*
GO:0019693	RIBOSE PHOSPHATE METABOLIC PROCESS	1.60	< 0.001*	− 1.12	0.181	2.15	< 0.001*
GO:0046390	RIBOSE PHOSPHATE BIOSYNTHETIC PROCESS	1.71	< 0.001*	− 1.03	0.423	2.18	< 0.001*

*SUV*_*max*_ maximum standardized uptake value, *PTMC* papillary thyroid microcarcinoma, *Macro-PTC* macro papillary thyroid carcinoma. *p value < 0.05.

## Discussion

In this study, we analyzed the clinicopathological characteristics of PTC^SUV-low^ and PTC^SUV-high^ patient groups and investigated their metabolic features using transcriptomic analysis. We found that SUV_max_ was positively correlated with tumor size, and the PTC^SUV-high^ group exhibited higher ECI and gross ETE rates than the PTC^SUV-low^ group after adjustments of tumor size. Transcriptomic analysis revealed lower expression of TDS genes in the PTC^SUV-high^ compared to the PTC^SUV-low^ group, and SUV_max_ was significantly associated with various gene signatures, including DNA replication, pyrimidine metabolism, purine metabolism, and Cell cycle. To determine the molecular characteristics that are independent of tumor size, a DEG analysis of the four tumor size and SUV_max_ subgroups identified five shared DEGs (upregulated PSG5, and downregulated *TFF3*, *SOX2*, *SLC5A5*, and *SLC5A7*) that were related to SUV_max_ and RFS, and three DEGs (upregulated *HOXD10* and downregulated *IFNA1* and *FER1L6*) related to tumor size and RFS that were unrelated to SUV_max_.

PET/CT plays an important role in the diagnosis, staging, and treatment response assessment of various solid cancers. In thyroid cancer, PET/CT scans are not routinely performed; instead, they are recommended in patients with an aggressive subtype and poorly differentiated thyroid cancer at initial staging and follow-up. Clinical practice is based on the inverse relationship between RAI-avidity and FDG-avidity^28^, however there was no study focused on the dissection of PET-CT imaging and transcriptomics in thyroid cancer. Tumors with high SUV_max_ are generally considered to have high glycolytic activity and the Warburg effect explains that aggressive tumors gain energy from aerobic glycolysis rather than from the TCA cycle, producing lactate than pyruvate^29^. In contrast to the previous study reporting a positive correlation between GLUT3 and GLUT4 protein expression and SUV_max_ in PTC^23^, our transcriptomics shows a negative or neutral relationship between GLUT family gene expression and SUV_max,_ although glycolysis tended to show a positive correlation with SUV_max_, suggesting GLUTs gene expression is not directly aligned with glycolysis.

The clinicopathological impact of high SUV_max_ tumors has been extensively investigated in various tumors^30,31^. In thyroid cancer, a previous retrospective study of a relatively small number of patients (N = 88) failed to show a difference in SUV_max_ between the recurrent and non-recurrent group^32^. However, another retrospective study with an 8-year follow-up period with a large patient size (N = 400) showed a significant survival decrease in patients with high-SUV_max_ determined on the initial PET/CT scan^21^. In agreement with a previous study, the PTC^SUV-high^ group in our cohort exhibited lower expression levels of TDS genes, and RFS was shorter in this group than in the PTC^SUV-low^ group. When we focused on PTMC, although ECI and gross ETE rates were higher in patients with PTMC^SUV-high^ than in those with PTMC^SUV-low^, we could not find a difference in recurrence, primarily due to the small number of patients in these subgroups. Therefore, further studies with larger numbers of patients are required to validate the efficacy of PET/CT scans in predicting recurrence in PTMC.

Tumor progression is accompanied by both the physical growth of the tumor and concomitant metabolic changes, making it challenging to identify the genes and pathways responsible for both these changes separately. Our study revealed five SUV_max_-related DEGs (*PSG5*, *TFF3*, *SOX2*, *SLC5A5*, and *SLC5A7*) contributing to RFS differences. The *PSG5* gene, upregulated in PTC^SUV-high^ regardless of tumor size, was previously reported as a prognostic marker for laryngeal cancer and is known to interact with prognostic lncRNAs in gastric cancer^33,34^. However, its prognostic role in thyroid cancer has not been determined. Another SUV_max_-related gene, *TFF3*, which is downregulated in PTC^SUV-high^ tumors, plays a role in angiogenesis and tumorigenesis in breast, stomach, and colon cancers. In thyroid cancer, low expression of *TFF3* can increase cell proliferation, migration, and invasion via activation of the IL-6/JAK/STAT3 signaling pathway^35^. These inflammatory pathways lead to high immune cell infiltration around thyroid cancer cells and could serve as a source of increased SUV_max_^36^. High expression of the stemness marker *SOX2* is associated with poor prognosis in several solid tumors and is a regulator of *GLUT1* expression^37,38^. In our cohort, the PTC^SUV-high^ group also exhibited low *SOX2* expression and relatively low levels of *GLUT1*^37,38^. *SLC5A5* is a well-known marker of thyroid differentiation, and PTC with low expression of *SLC5A5* is iodine non-avid and has a poor prognosis^39^. *SLC5A7*, which encodes a choline transporter, is downregulated in various solid cancers, and its expression is markedly suppressed in PTC^SUV-high^^40^. In colorectal cancer, promoter methylation and the resultant low expression of *SLC5A7* are poor prognostic factors as our results.

We also identified three DEGs (*HOXD10*, *IFNA1*, and *FER1L6*) related to tumor size. The expression of *HOXD10* is low in PTC, and the *HOXD10* gene is hypermethylated in BRAF^V600E^ mutants^41^. However, PTC^SUV-high^ in our study exhibited high expression levels of *HOXD10,* which might be related to large tumor size. *HOXD10* overexpression has been reported to induce cancer cell proliferation, while low expression induced invasion and metastases in head and neck cancer cell lines, supporting the proliferative role of *HOXD10* in cancer^42^. A previous study showed low expression of *FER1L6* in PTC; however, its prognostic significance and mechanism are not fully understood^43^. *IFNA1* has an antitumor effect that inhibits proliferation; thus, low expression of *IFNA1* could lead to cancer cell proliferation^44^.

Several studies have been conducted to understand the metabolic features of high SUV_max_ tumors using transcriptomic analyses. In breast cancer, the SUV-high-cluster was associated with frequent *TP53* mutations and enhanced the expression of downstream glycolysis genes through FOXM1-LDHA^45^. In multiple myeloma, a negative ^18^F-FDG PET/CT scan was associated with low expression of hexokinase-2, whereas a positive scan is accompanied by high expression of proliferation genes or *GLUT5*^46^. In intrahepatic cholangiocarcinoma, cell cycle processes, cell division, and mitosis gene sets were enriched in high SUV_max_ tumors^47^. Similarly, in this study, the PTC^SUV-high^ group exhibited enriched gene sets for DNA replication, cell cycle processes, and ribosome assembly, regardless of tumor size, in both KEGG and GOBP analyses. Notably, some gene sets showed differences in SUV_max_ in macro-PTC but not in PTMC: these were the gene sets for glucose import, glycolysis, citrate cycle TCA cycle, purine metabolism, one-carbon pool by folate, and the pentose phosphate pathway. For tumor growth or proliferation, new macromolecules, such as nucleic acids, lipids, and proteins, are essential, and macro-PTC reprograms and exploits cellular pathways to obtain the materials necessary for proliferation. These cellular pathways could be targets for anticancer therapy, and further studies are needed to assess the precise manipulation of key steps.

In summary, we investigated the clinicopathological and transcriptomic features of PTC based on the SUV_max_ and tumor size. In PTMC and PTC, tumors with high SUV_max_ exhibited more capsular invasion and gross ETE than low SUV_max_ tumors. DEG analyses revealed the genes contributing to RFS and related to SUV_max_ (*PSG5*, *TFF3*, *SOX2*, *SLC5A5*, and *SLC5A7*) and tumor size (*HOXD10*, *IFNA1*, and *FER1L6*). GSEA revealed that gene sets for DNA replication, cell cycle processes, and ribosome assembly were enriched in high SUV_max_ tumors regardless of tumor size, whereas gene sets for glucose import, glucose metabolic process, purine metabolism, and the pentose phosphate pathway were related to large tumor size.

Our research provides insight into metabolic reprogramming of PTC related to SUV_max_, as well as markers to account for SUV_max_ and tumor size-related RFS. Going beyond the current method of evaluating tumors only by size, using suggested gene biomarkers as well as SUV_max_ to classify tumors into more diverse subgroups will help predict patient prognosis and pave the way for tailor-made treatment protocols in the future.

## Materials and methods

### Study population

We retrospectively reviewed 80 patients postoperatively diagnosed with PTC who underwent preoperative ^18^F-FDG-PET/CT and provided informed consent for collection of fresh frozen thyroid tissue from January 2003 to December 2010. Prior to the 2015 ATA guidelines, total thyroidectomy was performed in patients with the tumor size of 1 cm or more, bilateral multifocality, aggressive variant type, ETE in preoperative radiology (except ETE to only the strap muscle), or N1b lymph node metastasis. 70 patients were received total thyroidectomy and 10 patients were received lobectomy. Among the 10 patients, none underwent recurrence or completion thyroidectomy. All patients who underwent total thyroidectomy were received radioactive iodine (RAI) treatment according to 2009 ATA guideline. All patients were denied the history of diabetes related with glucose signaling. Data were retrospectively collected, including demographic information, laboratory findings, SUV_max_, and pathology data. This study was approved by the Institutional Research and Ethics Committee at Chungnam National University Hospital (CNUH-2022-11-004-001) and conducted in accordance with the Declaration of Helsinki. Informed consent was obtained from all individual participants involved in the study. All personal identifiers were removed or disguised to ensure participant anonymity, in line with HIPAA guidelines.

### Postoperative follow-up and recurrence

Patients were followed up for 8.8 ± 0.5 years (mean ± SEM). After primary treatment, all patients underwent TSH suppression therapy with thyroid hormone supplementation according to the American Thyroid Association guidelines^2^. Patients were assessed every 3 months in the first year after surgery, every 6 months for the next 2 years, and annually thereafter. Thyroid ultrasound imaging and thyroid function tests (including thyroglobulin and antithyroglobulin antibodies), were routinely performed at each follow-up consultation. Indeterminate or suspicious thyroid nodules or lymph nodes (LNs) were evaluated by fine-needle aspiration. All six structural recurrence cases were confirmed by cytological analysis, two from the operation bed and four from the lateral LNs. One patient died due to airway obstruction due to the tumor extending significantly into the mediastinum, while the remaining five patients were cured and maintained stable disease after treatment with I-131 100–150 mCi RAI or neck dissection.

### RNA extraction for sequencing

To analyze the transcriptome and identify DEGs, RNA was extracted from tumor and paired non-tumor tissue samples. Thyroid samples were isolated from specimens frozen at − 80 °C immediately after thyroidectomy and homogenized using a mortar and pestle; total RNA was extracted using an RNA extraction kit (QIAGEN, Germantown, MD, USA) following the manufacturer’s protocol. All experiments were conducted under clean conditions and equipment was pre-autoclaved. The quality of the extracted RNA was evaluated using the Agilent 2100 Bioanalyzer RNA Nano Chip (Agilent, Santa Clara, CA, USA). The extracted RNA was used to construct RNA libraries using the TruSeq access library or the stranded mRNA LT Sample Preparation Kit (Illumina, San Diego, CA, USA), according to the manufacturer’s protocols. Library quality was analyzed using an Agilent 2100 Bioanalyzer and an Agilent DNA 1000 kit (Agilent, Santa Clara, CA, USA). Samples were sequenced on the Illumina HiSeq 2500 platform (Illumina, San Diego, CA, USA), yielding an average of 38 million paired-end 100 bp reads.

### Bioinformatic transcriptome analysis

To analyze the relationship between the thyroid differentiation score (TDS) and SUV_max_, we used the “ComplexHeatmap” and “corrplot” R packages with log10 transcript per million (TPM). We calculated the TDS by combining the gene set variation analysis (GSVA) package with the TDS gene list^15^. To confirm survival probability, we used the “survival” and “survminer” R packages. Additionally, we identified DEGs by subdividing our cohort into two groups based on tumor size and SUV_max_. Differential expression analysis was carried out in R using “DESeq2” and enrichment analysis was performed using the “fgsea” R package. Gene sets used for Kyoto Encyclopedia of Genes and Genomes (KEGG) pathway and Gene Ontology enrichment analyses were downloaded from the Gene Set Enrichment Analysis (GSEA) website (https://www.gsea-msigdb.org). DEGs and KEGG pathways with corrected *p* values < 0.05 were considered statistically significant. We assessed RFS by specific gene expression level using open database from GEPIA 2 which is based on TCGA database.

### Statistical analysis

Group data for continuous variables are presented as mean ± standard deviation and, in some cases, as mean ± standard error of the mean, as noted in the footnotes. Categorical variables were presented as numbers and percentages. To compare the means of continuous variables, we used the unpaired Student’s *t*-tests or Mann–Whitney *U* test. Chi-square tests or Fisher’s exact test were used to compare the distributions of categorical variables, and Pearson’s correlation analysis was used to evaluate the associations between tumor size and SUV_max_. Kaplan–Meier (K–M) survival curves were created to evaluate differences in RFS between the groups, and receiver operating characteristic (ROC) curves were used to determine the cutoff SUV_max_ value for predicting recurrence. For tumor size- or SUV_max_-adjusted statistical analyses, we used ANCOVA, Mantel–Haenszel Chi-square test, and Cox regression analysis. Statistical significance for all analyses was established with a two-tailed *p* value < 0.05. Statistical analyses/graphs were performed/created using SPSS Version 26.0. (IBM corp., Armonk, NY, USA), R, GraphPad Prism 9.4.1. (GraphPad Software Inc., San Diego, CA, USA), and OriginPro 2021 (OriginLab Corp., Northampton, MA, USA).

### Supplementary Information

Supplementary Figure 1.

Supplementary Figure 2.

Supplementary Figure 3.

Supplementary Legends.

Supplementary Tables.

## Data Availability

The accession number for the bulk RNA sequencing dataset is GSE213647 (secure token: avwpkueoznwdzux).
