# Supplementary figures and images for: Transcriptomic characteristics according to tumor size and SUVmax in papillary thyroid cancer patients
Source: Sci Rep. 2024 May 14;14:11005. doi: 10.1038/s41598-024-61839-0 (PMC11094162; doi:10.1038/s41598-024-61839-0)

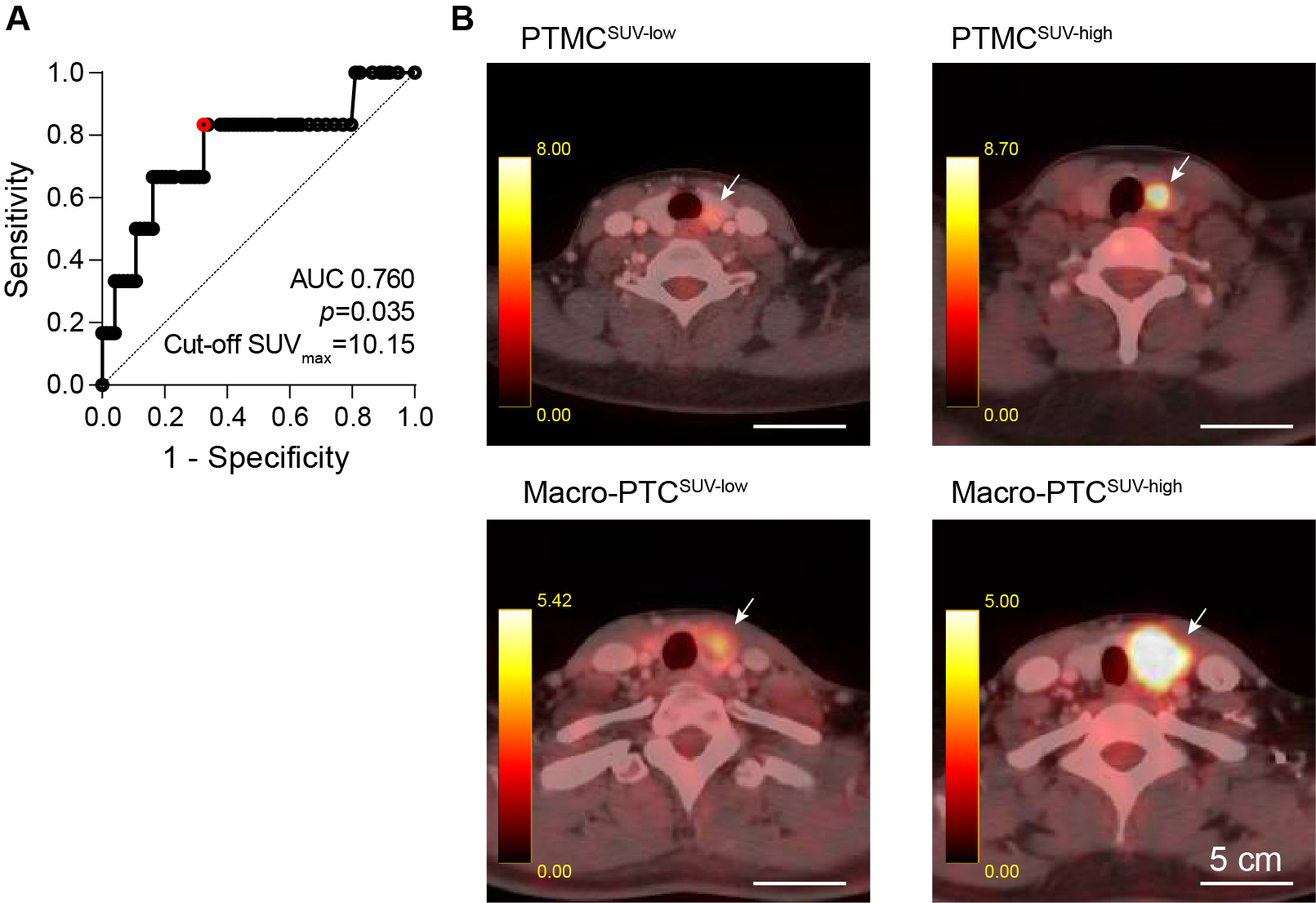

Supplement: Supplementary file 1 — Supplementary Figure 1. [file 41598_2024_61839_MOESM1_ESM.jpg]

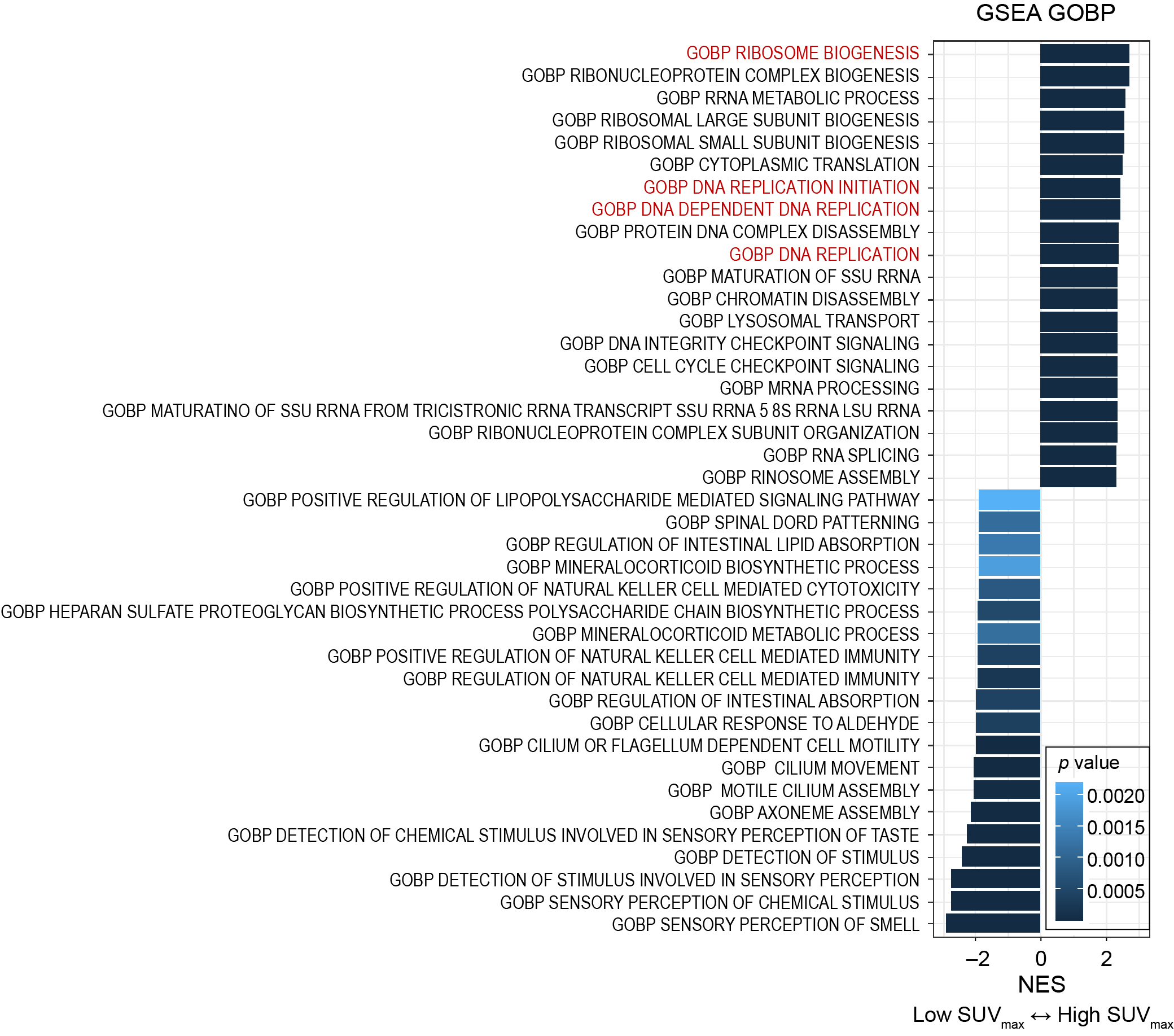

Supplement: Supplementary file 2 — Supplementary Figure 2. [file 41598_2024_61839_MOESM2_ESM.jpg]

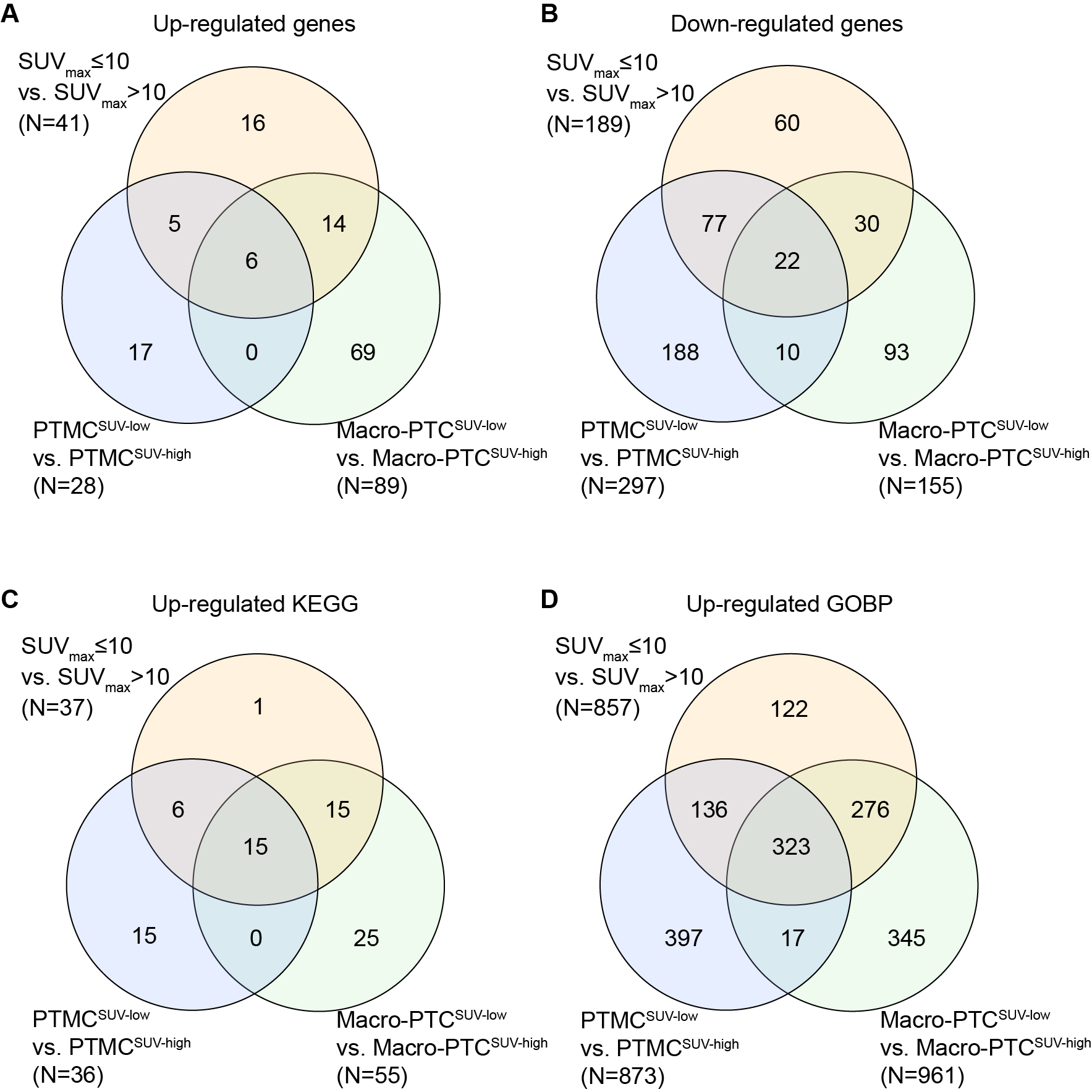

Supplement: Supplementary file 3 — Supplementary Figure 3. [file 41598_2024_61839_MOESM3_ESM.jpg]
