# Supplementary material for: Transcriptomic characteristics according to tumor size and SUVmax in papillary thyroid cancer patients
Source: Sci Rep. 2024 May 14;14:11005. doi: 10.1038/s41598-024-61839-0 (PMC11094162; doi:10.1038/s41598-024-61839-0)
Supplement: Supplementary file 4 — Supplementary Legends. [file 41598_2024_61839_MOESM4_ESM.docx]

**Supplementary figure 1. Post-operative recurrence prediction with SUV_max_ from pre-operative 18F-FDG PET/CT scan in PTC patients and their representative images.**

(**A**) ROC curve for prediction of the post-operative recurrence from preoperative SUV_max_ (AUC = 0.760, *P* = 0.035, and Cut-off SUV_max_ = 10.15). (**B**) PET/CT scan images showing SUV_max_ of PTMC^SUV-low^, PTMC^SUV-high^, Macro-PTC^SUV-low^, and Macro-PTC^SUV-high^ tumors. In PTMC^SUV-low^ tumor, SUV_max_ was 3.8 and tumor size was 0.7 cm. In PTMC^SUV-high^ tumor, SUV_max_ was 10.2 and tumor size was 0.7 cm. In Macro-PTC^SUV-low^ tumor, SUV_max_ was 3.5 and tumor size was 1.7 cm. In Macro-PTC^SUV-high^ tumor, SUV_max_ was 12.9 and tumor size was 3.3 cm. Tumors are indicated with white arrow and SUV_max_ scale bar is shown in the left side of each images. The scale bar is 5 cm.

**Supplementary figure 2.** Comparison of GSVA based on unsupervised analysis using Gene Ontology analysis between PTC^SUV-low^ and PTC^SUV-high^ tumors.

**Supplementary figure 3. Relationship of DEGs elicited from comparisons by SUV_max_ and tumor size.**

(**A-B**) Venn diagram showing distribution of upregulated (**A**) and down-regulated (**B**) DEGs from analyses; SUV_max_ ≤ 10 vs. > 10, PTMC^SUV-low^ vs. PTMC^SUV-high^, Macro-PTC^SUV-low^ vs. Macro-PTC^SUV-high^. (**C-D**) Venn diagram showing distribution of upregulated KEGG (**A**) and upregulated GOBP (**B**) from analyses; SUV_max_ ≤ 10 vs. > 10, PTMC^SUV-low^ vs. PTMC^SUV-high^, Macro-PTC^SUV-low^ vs. Macro-PTC^SUV-high^.
